# Supplementary material for: Physical embodiment enables information processing beyond explicit flow sensing in active matter
Source: Sci Adv. 2026 Mar 13;12(11):eaec0783. doi: 10.1126/sciadv.aec0783 (PMC12985729; doi:10.1126/sciadv.aec0783)
Supplement: Supplementary file 1 — Sections S1 to S8 Figs. S1 to S7 Table S1 Legends for movies S1 to S4 [file sciadv.aec0783_sm.pdf]

Science Advances
AAAS

Supplementary Materials for

Physical embodiment enables information processing beyond explicit flow sensing in active matter

Diptabrata Paul *et al.*

Corresponding author: Frank Cichos, cichos@physik.uni-leipzig.de

Sci. Adv. **12**, eaec0783 (2026)
DOI: 10.1126/sciadv.aec0783

The PDF file includes:

Sections S1 to S8
Figs. S1 to S7
Table S1
Legends for movies S1 to S4

Other Supplementary Material for this manuscript includes the following:

Movies S1 to S4

S1 Experimental setup

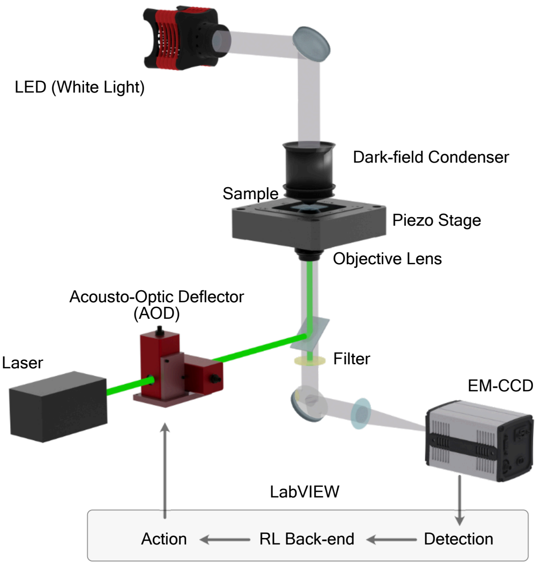

Figure S1: Schematic of the setup used for the experiments

S2 Hydrodynamic flow field control

The hydrodynamic flow field was generated by harnessing the thermo-plasmonic property of thin gold film ($50\,\mathrm{nm}$), deposited on the bottom coverslip of the sample chamber. Tightly focusing laser of $532\,\mathrm{nm}$ wavelength leads to absorption ($\sim 30\%$) and consequent conversion to heat which perturbs the solid-liquid interactions. Such local temperature perturbations of the solid–liquid interactions at the interface induces thermo-osmotic(TO) flow , given

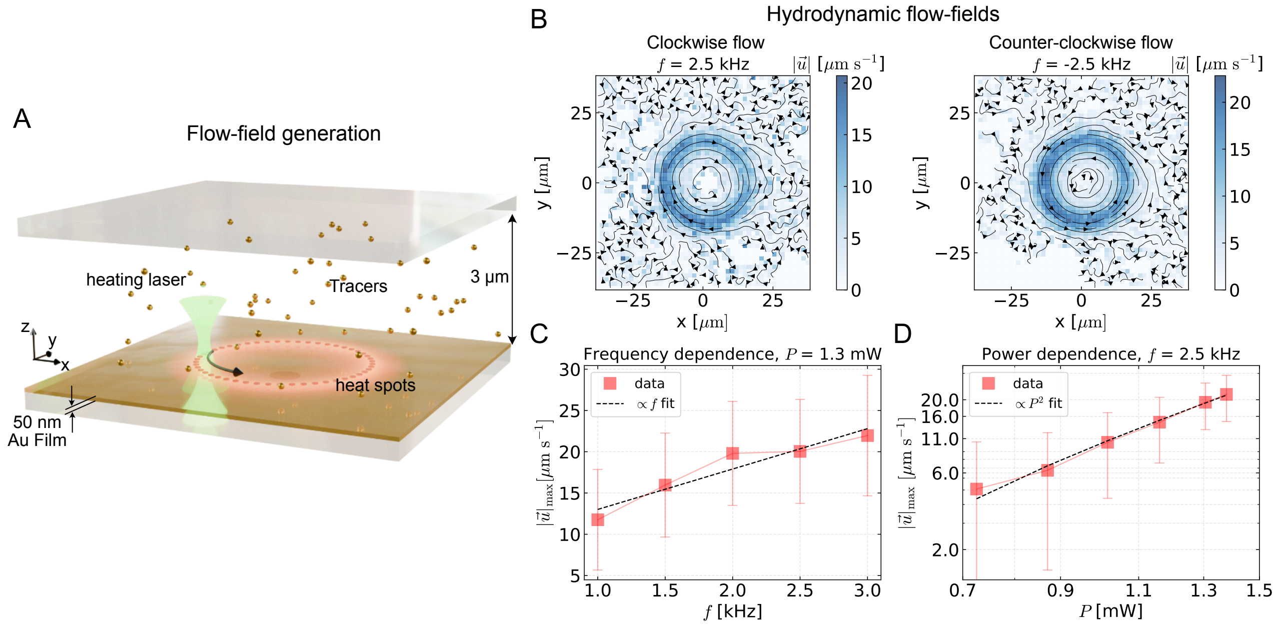

Figure S2: **Realization of hydrodynamic flow field.** **A**. The hydrodynamic flow field is generated by periodically heating a thin ($50\,\mathrm{nm}$) Au film at discrete heat-spots indicated by red dots arranged on the circumference of a circle of radius $15\,\mu\mathrm{m}$. The resulting flow is traced using $250\,\mathrm{nm}$ AuNP tracers. **B**. flow field rotating along clockwise or counter-clockwise direction is generated by altering the laser scanning frequency to either $f = 2.5\,\mathrm{kHz}$ or $f = -2.5\,\mathrm{kHz}$ respectively at laser power $P = 1.30\,\mathrm{mW}$. **C**. The thermo-viscous (TV) flow speed scales linearly with frequency ($\propto f$) of the thermal wave generated by the laser scanning. **D**. The TV flow field scales quadratically with temperature increment in the gold film (ΔT), therefore also scales quadratically with the incident laser power ($\propto \Delta T^2 \propto P^2$).

by:

$$u_{\text{TO}} = -\frac{1}{\eta}\int_0^\infty z\, h(z)\frac{\nabla_\parallel T}{T}dz = \chi\frac{\nabla_\parallel T}{T} \tag{1}$$

Here, η is the viscosity of the liquid, $h(z)$ is the excess enthalpy, z being the interface normal axis, T is the temperature, $\nabla_\parallel T$ is the temperature gradient parallel to the surface and $\chi \sim 10 \times 10^{-10}\mathrm{m}^2\,\mathrm{s}^{-1}$ represents the thermo-osmotic coefficient, leading to the liquid being driven towards the hotter region in the boundary layer.
In addition, scanning the laser spot at high frequencies ($f \geq 1\,\mathrm{kHz}$) and elevated powers ($P \geq 0.5\,\mathrm{mW}$) induces additional fluid flow, arising from mass transport driven by thermal expansion and the temperature-dependent viscosity of water, as shown in Fig. S2(a). This thermo-viscous (TV) flow is typically directed in the opposite direction to the scanning induced thermal-wave of amplitude ΔT, and is given by:

$$u_{\text{TV}} = -\frac{3\sqrt{\pi}}{4}f\alpha\beta w\Delta T^2 \tag{2}$$

Here, $\alpha = (1/\rho)(\partial\rho/\partial T)$ represents the thermal expansion coefficient and $\beta = (1/\eta)(\partial\eta/\partial T)$ represent the temperature dependence coefficient of the viscosity. For water, using typical values of thermal expansion coefficient $\alpha = 3.3 \times 10^{-4}\ \mathrm{K}^{-1}$ and temperature-dependent viscosity coefficient $\beta = 0.021\ \mathrm{K}^{-1}$, a laser beam width of $w \approx 0.8\,\mu\mathrm{m}$, modulation frequency $f = 2.5\,\mathrm{kHz}$, and temperature increase $\Delta T \approx 35\,\mathrm{K}$ at laser power $P = 1.30\,\mathrm{mW}$, the resulting flow speed is estimated to be $|\vec{u}|_{\text{max}} \approx 23\,\mu\mathrm{m\,s}^{-1}$.

Flow field measurement: The microswimmer is subjected to a combined flow-perturbation: $\vec{u} = \vec{u}_{\text{TO}} + \vec{u}_{\text{TV}}$; however, in the bulk fluid, the TV contribution dominates the overall flow dynamics. The flow field is traced by introducing $250\,\mathrm{nm}$ gold nanoparticles and analyzing the tracked trajectories as shown in Fig. S2A. The resulting flow fields generated at a laser power of $P = 1.30\,\mathrm{mW}$ with scanning frequencies $f = 2.5\,\mathrm{kHz}$ and $f = -2.5\,\mathrm{kHz}$ are shown in Fig. S2B, corresponding to clockwise and counter-clockwise flows respectively. The resulting maximum flow speed ($|\vec{u}|_{\text{max}}$) depends linearly with frequency (f), as shown in Fig. S2C and exhibits quadratic dependency on the temperature increment (ΔT) and consequently the laser power(P), illustrated in Fig. S2D.

Dynamic flow field generation:
Time-varying flow fields were generated by scanning a focused laser beam along a spline trajectory. The spline is generated by interpolating between six control nodes whose positions oscillate on circular paths around fixed centers according to $\vec{r}_i(t) = \vec{c}_i + R_i\big[\cos(\omega_i t),\, \sin(\omega_i t)\big]$. Here $\vec{c}_i$ are the centers, R_i the radii, and ω_i the individual angular frequencies of the nodes. The trajectory is initialized by randomly generating six control nodes in a predefined spatial range that define its initial shape having random oscillation radius ($R_i \in [3.8, 7.5]\,\mu\mathrm{m}$) and angular frequencies ($\omega_i \in [0.5, 1.0]\,\mathrm{rad\,s}^{-1}$). This stochastic initialization ensures that each realization of the flow field exhibits a unique, non-repetitive temporal evolution, thereby capturing the dynamic and unpredictable nature of real microfluidic environments.
At each instant, the node coordinates are used to construct a closed cubic B-spline, which is then scanned by the laser at $f = -2.5\,\mathrm{kHz}$. This frequency being much higher than the node motion leads to a time-averaged flow field ($\vec{u}$) corresponding to the instantaneous spline shape. As the node positions evolve, the resulting flow field changes smoothly in both space and time, providing a controlled dynamic environment for the learning experiments. Fig. S3 shows three instantaneous spline configuration (red-dots) from the temporal evolution of the perturbation as well the corresponding measured flow field when scanned at frequency $f = -2.5\,\mathrm{kHz}$. The corresponding scanning direction is indicated by the red-arrows.

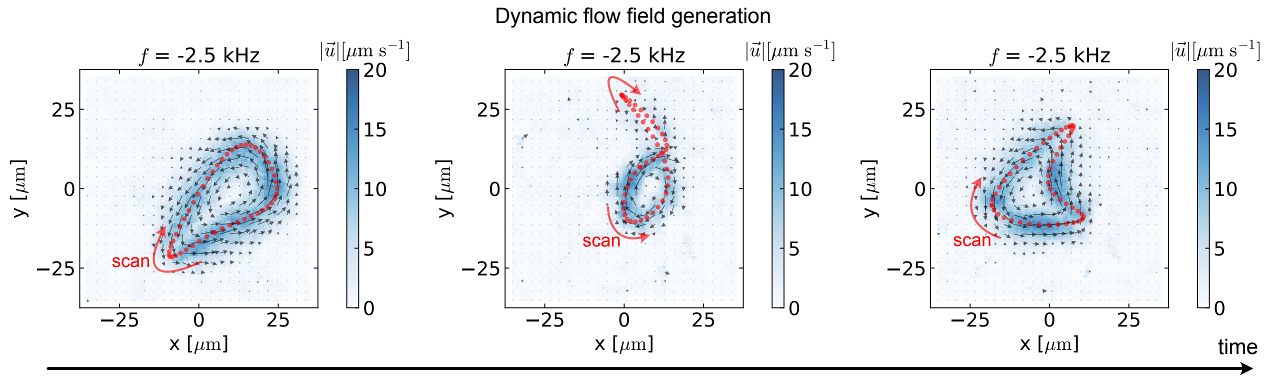

Figure S3: **Dynamic flow field generation.** The flow field is produced by continuously varying the laser scanning (frequency $f = -2.5\,\mathrm{kHz}$) trajectory, represented by a spline curve (red dots) interpolated between six control nodes. The red arrows indicate the instantaneous scanning direction, while the quiver plot illustrates the corresponding measured flow field generated by the instantaneous configuration of the evolving pattern.

S3 Reinforcement learning algorithm

The set of actions ($\mathcal{A} : \{a_{\uparrow}, a_{\downarrow}, a_{\leftarrow}, a_{\rightarrow}, a_{\circ}\}$) and the reward defined (r_t) in the main text allows the agent to interact with the environment and learn strategies under different environmental conditions. The strategy is represented by a policy $\pi(a_t \mid s_t)$ at a time step t and defines a probability distribution over actions conditioned on the current state s_t, from which an action a_t is sampled. Over the course of many episodes, the agent will update the policy to maximize the expected cumulative reward.

For our experiments, we have chosen the proximal policy optimization (PPO) algorithm, an RL algorithm based on the actor-critic framework, where the actor (policy) and the value function (critic) are jointly optimized. The central idea of PPO is to prevent large deviation from the previous policy by using a clipped objective function $L^{\text{CLIP}}(\phi)$:

$$L^{\text{CLIP}}(\phi) = \mathbb{E}\big[\min\big(R_t(\phi)\hat{A}_t,\ \text{clip}(R_t(\phi),\ 1-\varepsilon,\ 1+\varepsilon)\hat{A}_t\big)\big]. \tag{3}$$

$\mathbb{E}$ denotes the empirical expectation over states and actions in sampled trajectories. ϕ is the policy parameter, $R_t(\phi) = \frac{\pi_\phi(a_t|s_t)}{\pi_{\phi_{\text{old}}}(a_t|s_t)}$ is the ratio of the policy under the new and old parameters, ε is exploration governing policy clip hyperparameter. $\hat{A}_t$ is the generalized advantage estimation (GAE) function at time t, that reduce variance in the policy gradient estimate, and is given by:

$$\hat{A}_t = \delta_t + (\gamma\lambda)\delta_{t+1} + \cdots + (\gamma\lambda)^{T-t+1}\delta_{T-1}. \tag{4}$$

$$\text{where, } \delta_t = r_t + \gamma V(s_{t+1}) - V(s_t) \tag{5}$$

Here, $t \in [0, T]$ specifies the step index, λ is the GAE decay parameter controlling the trade-off between bias and variance. The value function $V(s_t)$ is updated using the temporal difference (TD) error δ_t, and γ is the discount factor hyperparameter, determining how strongly future rewards influence the present update. The learning rate α determines the step size of the parameter updates for both networks, chosen to balance convergence speed and stability in the presence of experimental fluctuations. In PPO based framework, while the reward is computed and assigned at each iteration step based on the agent's instantaneous state transition (step-wise reward), policy is optimized via batch updates (given by batch size) to maximize the expected cumulative reward (return) over the episode. Thus long-term expected return is realized via periodic policy improvements.

The following table (table S1) contains additional details and parameters:

Parameters	Policy Network	Value Network	Activation	Batch Size	α	γ	ε	GAE λ
Values	$[128, 128]$	$[128, 128]$	ReLU	10	2×10^{-4}	0.95	0.1	0.95

Table S1: Additional parameters of the RL program

S4 Policy investigation for inert environment

In course of navigation, the action is chosen by sampling a probability distribution of the discrete action set $\mathcal{A} : \{a_{\uparrow}, a_{\downarrow}, a_{\leftarrow}, a_{\rightarrow}, a_{\circ}\}$. The first four actions correspond to active propulsion in the respective directions, while the fifth action, $a_\circ$, relies solely on passive translational diffusion for navigation. To analyze, the saved actor model corresponding to a given training stage was loaded, and the spatial domain was discretized into a 100×100 grid of positions to construct states out of normalized coordinates $\{x_i, y_i, \Delta d\}$ ($\Delta d = 1/100$). For each grid point, the trained actor network saved at a certain step was inquired to obtain the spatial action probability distribution $p_{a_i} = \pi(a_i|s_i)$. Fig. S4 A shows the spatial probability distribution for the actions ($\mathcal{A}$) of an agent trained for 50 episodes in an inert environment. The target position is indicated by the green circle. The distribution indicates that probabilities for selecting an action increases with higher distances from the target. Additionally, the probability of selecting $a_\circ$ remains low due to the reward structure favoring active movement toward the target.

The resulting inert policy $\langle \vec{v}_{\text{A}} \rangle_{\text{inert}}$ therefore resembles a radially inward velocity field with progressive training. The corresponding sink position shifts with training (indicated by the gradient color in Fig. S4B (left)) and eventually overlaps with the target position, indicated by the green circle. The distance d_s between the policy sink and target position decreases with training steps as shown in Fig. S4B (right), and eventually aligns very closely with the pre-defined threshold distance d_{th} (horizontal dashed line). The probability distribution of d_s extracted from the last 3000 steps (shaded region) yields a mean value of $d_s = 2.34 \pm 0.86$ µm (see inset), quantifying the target acquisition precision and accuracy.

The action probabilities p_{a_i}, obtained from the learned policy in an inert environment, can also be used to compute a weighted average of the experimentally measured velocity fields $\vec{v}_i^{\text{exp}}$ associated with each discrete action in the inert environment. This yields the expected velocity field $\vec{v}_{\text{inert}}^{\text{exp}} = \sum_{a_i \in \mathcal{A}} p_{a_i} \vec{v}_i^{\text{exp}}$, which as shown in Fig.S4C, closely resembles an ideal radially inward vector field. The convergent behavior is further showcased by the rapid increase of the episodic reward (Fig. S4D) with progressive training and demonstrates effective policy learning.

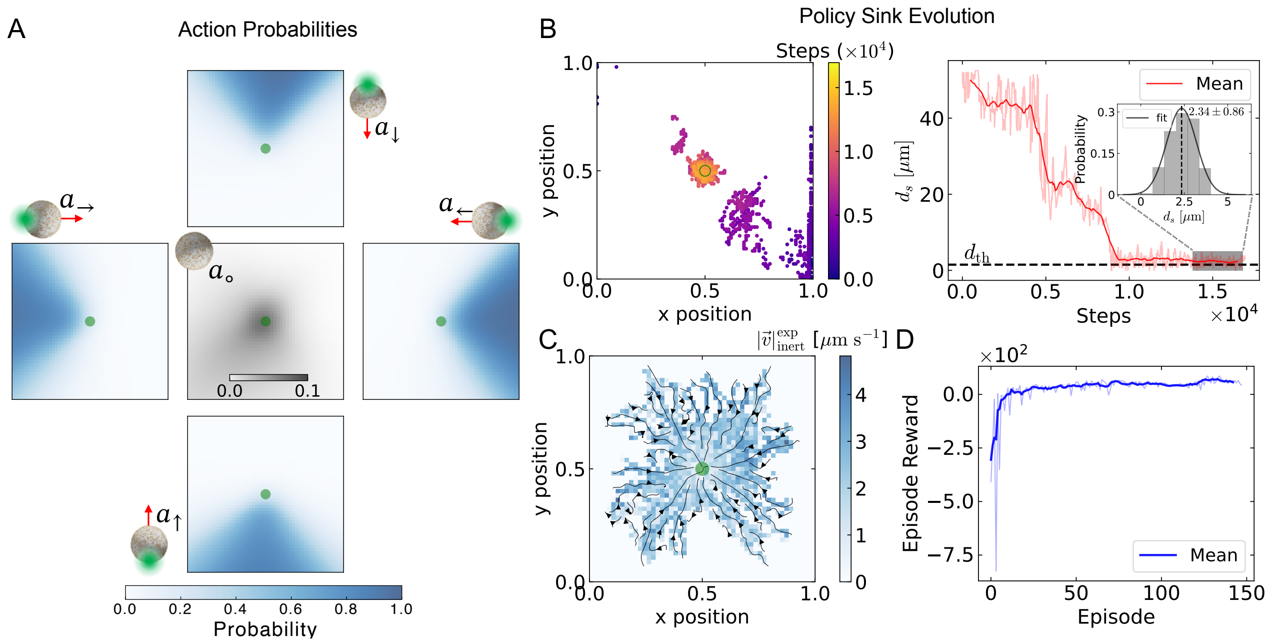

Figure S4: **Investigation of inert-policy. A**. The spatial probability distribution of the actions $\{a_\uparrow, a_\downarrow, a_\leftarrow, a_\rightarrow, a_\circ\}$ after 50 training episodes in an inert environment at average propulsion speed $v = 5.8\,\mu\text{m}\,\text{s}^{-1}$. Green circle indicates the target. **B** (left). Evolution of the sink position with progressive training steps (indicated by the gradient color) in an inert environment. The target position is indicated by the green circle. (right). The policy sink distance to the target position (d_s) decreases with increasing training steps. The threshold distance (d_{th}) is indicated by the horizontal dashed black line. (Inset) The probability distribution of converged d_s is extracted from the last 3000 exploration steps (shaded region) and leads to mean $d_s = 2.34 \pm 0.86$ µm. **C**. Expected velocity field $\langle \vec{v}_{\text{A}} \rangle^{\text{exp}}_{\text{inert}}$ obtained from the experimentally observed velocity components ($\vec{v}_i^{\text{exp}}$ for action a_i ($a_i \in \mathcal{A}$)) of the agent in an inert environment exhibits an radial profile. **D**. The net episodic reward increases rapidly with training episodes and stabilizes at an higher value, indicating convergence of the learning process.

S5 Propulsion speed dependence

One of the key feature of any microscopic agents such as the microswimmer or microorganism is the influence of Brownian motion, which is absent in macroscopic agents such as robots. It is common to quantify the effect of Brownian noise through a dimensionless quantity, Péclet number Pe $= Rv/D$, comparing the effect of deterministic motion (self propulsion speed v) over a characteristic length scale R (particle radius) to the diffusive motion characterized by the diffusion constant $D = 0.21\,\mu\text{m}^2\,\text{s}^{-1}$.
The positional noise can be controlled via changing the heating laser power on the particle, and the corresponding variation of the velocity exhibits a non-linear dependence as shown in Fig. S5A. The non-linearity is the result of the instantaneous particle slip motion within the exposure time $\tau \approx 160$ ms of the fixed heating laser. The speed in turn influences the angular distribution of the displacement of the agent, which as expected is higher for lower speeds and vice versa, as shown in Fig. S5A. The corresponding Péclet number in our experiments ranged from 18 to 35. The differences in agent's policies at various propulsion speeds can be analyzed by comparing them at a fixed time point during convergence. This comparison is shown in Fig. S5B, taken after approximately 8000 steps in the environment for propulsion speeds $v = 3.7\,\mu\text{m}\,\text{s}^{-1}$, $v = 4.6\,\mu\text{m}\,\text{s}^{-1}$, and $v = 5.8\,\mu\text{m}\,\text{s}^{-1}$. The corresponding $\langle \vec{v}_{\text{A}} \rangle_{\text{inert}}$, visualized by the black streamlines, are more prominently radial for agent trained with higher propulsion speed. Concurrently, the corresponding entropy change (ΔH) for the agents operating at higher speeds converge to policies with lower uncertainty, indicating greater confidence in action selection. As a result, the convergence time (τ_c) for agent with different propulsion speed vary significantly, as shown in Fig. S5C. This is obtained by fitting the episode length data to an exponential decaying function $\propto \exp -t/\tau_c$, shown by the dashed line in each of the plots for different speeds. For agents with speed $v = 3.7\,\mu\text{m}\,\text{s}^{-1}$, its $\tau_c = 20.67$ episodes ($\approx 7525 \pm 816$ steps) is almost twice as high as that for agent with speed $v = 5.8\,\mu\text{m}\,\text{s}^{-1}$, whose $\tau_c = 8.95$ episodes ($\approx 4118 \pm 600$ steps), where as agent with speed $v = 4.6\,\mu\text{m}\,\text{s}^{-1}$ converges at approximately $\tau_c = 16.86$ episodes ($\approx 5500 \pm 400$ steps). All these could be attributed to higher noise at lower propulsion speeds, leading to lower correlation of the action of the agent in the environment, resulting higher uncertainty and increased convergence time.

S6 Policy investigation for flow-perturbed environment

The hydrodynamic interaction of symmetric microswimmer agents in presence of the flow-perturbation leads to embodied dynamics. Such dynamics consists of the net effect $\vec{v}^{\text{exp}}_{a_i,\text{net}}$ resulting from the net effect of the agent's

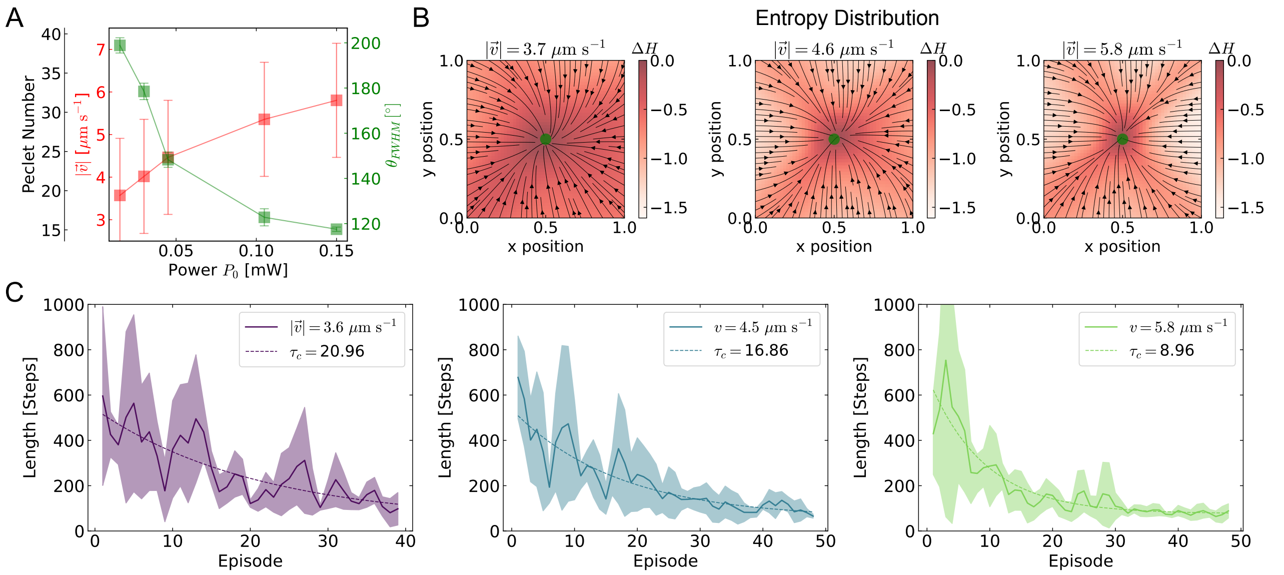

Figure S5: **Propulsion speed dependence.** **A**. Extracted microswimmer speed and the corresponding angular distribution FWHM (θ_{FWHM}) of the displacement vector as a function of heating power. The corresponding Péclet number varies between $[18, 35]$. **B**. After ~ 8000 exploration steps, the policy characterized by the entropy difference (ΔH) from the initial state shows that agents trained at higher propulsion speeds develop more deterministic navigation strategies with more prominant radial expected velocity field. The target position is marked by the green circle. **C**. The convergence rate, quantified by the decaying of training episode lengths decreases with increasing speed of the microswimmer agent. The corresponding decaying behavior is fitted to an exponential decay function ($\propto \exp(-t/\tau_c)$, where τ_c is convergence time constant).

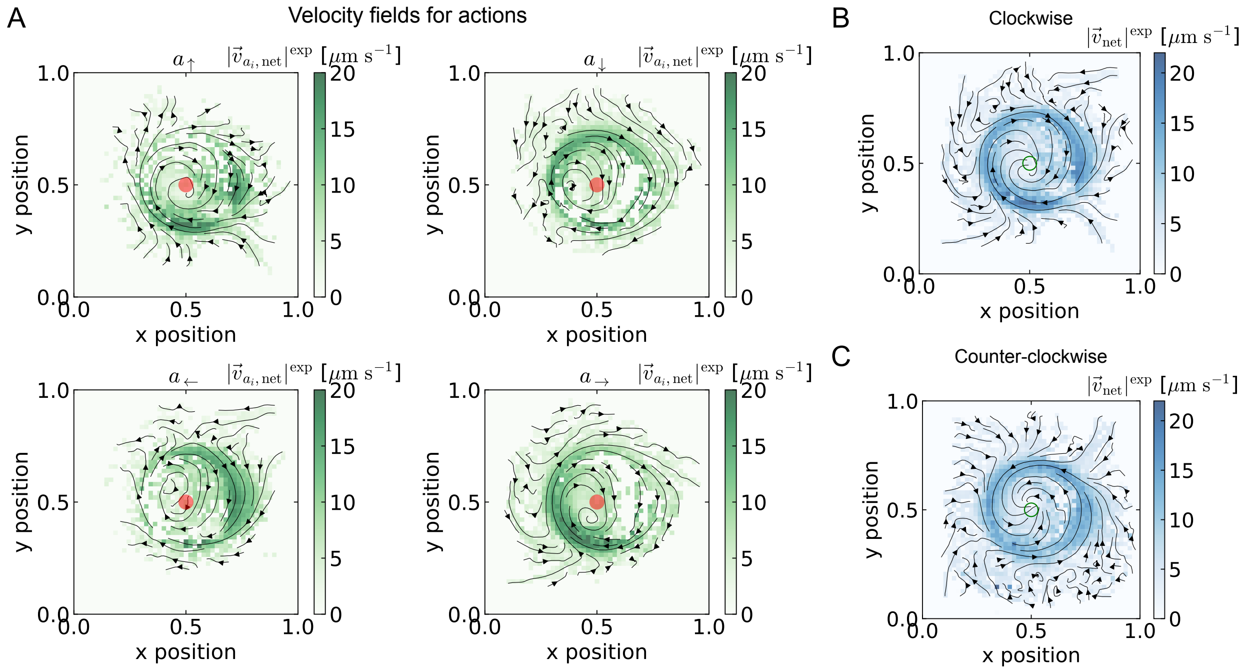

Figure S6: **Velocity and policy in flow field environment.** **A**. The velocity field distribution ($\vec{v}^{\,\mathrm{exp}}_{a_i,\mathrm{net}}$) obtained from the training trajectories in perturbed environment comprising clockwise flow-perturbation for the actions $a_\uparrow$, $a_\downarrow$, $a_\leftarrow$, $a_\rightarrow$. Target position is indicated by the red circle. **B**. Corresponding net velocity field $\vec{v}^{\,\mathrm{exp}}_{\mathrm{net}}$ for clockwise flow-perturbation obtained from combining the agent's velocity fields for each actions $\vec{v}^{\,\mathrm{exp}}_{\mathrm{net}} = \sum_{a_i \in \mathcal{A}} \vec{v}^{\,\mathrm{exp}}_{a_i,\mathrm{net}} \cdot p_{a_i}$, where p_{a_i} represent the corresponding probabilities from the policy. **C**. The net velocity field $\vec{v}^{\,\mathrm{exp}}_{\mathrm{net}}$ for a counter-clockwise flow-perturbation exhibit similar vortexial pattern, obtained from the experimental training trajectories and corresponding $\vec{v}^{\,\mathrm{exp}}_{a_i,\mathrm{net}}$.

action along with the clockwise flow-perturbation is decomposed from the training trajectories of the agent in the experimentally observed data, as shown in Fig. S6A. While away from the flow-perturbed region, the velocity field correspond to the predefined action directions, the strong flow advection leads to significant deviation of the motion.

The correlation of this net effect along with the corresponding probability, enables morphological computation from the proprioceptive cues provided to the agent.
Combination of the action probabilities (p_{a_i}) with $\vec{v}^{\text{exp}}_{a_i,\text{net}}$ allows us to compute the net velocity field of the agent from the experimental data $\vec{v}^{\text{exp}}_{\text{net}} = \sum_{a_i \in \mathcal{A}} p_{a_i} \cdot \vec{v}^{\text{exp}}_{a_i,\text{net}}$, as shown in Fig. S6B, for a clockwise flow field. The emerging vortexial structure around the target position (green circle) indicates successful navigation. Furthermore, the equivalence with the $\vec{v}_{\text{net}} = \langle \vec{v}_{\text{A}} \rangle_{\text{flow}} + \vec{u}$, derived in Fig. 3B in the manuscript, elucidates the embodied learning of the agent. Similarly, the net effect $\vec{v}^{\text{exp}}_{\text{net}}$ for the counter-clockwise flow perturbation exhibits a vortexial structure around, equivalent to the one shown in Fig. 4A in the manuscript. These results exemplify the embodied learning process of the agent in presence of the strong flow perturbation.

S7 Policy investigation for dynamic flow field

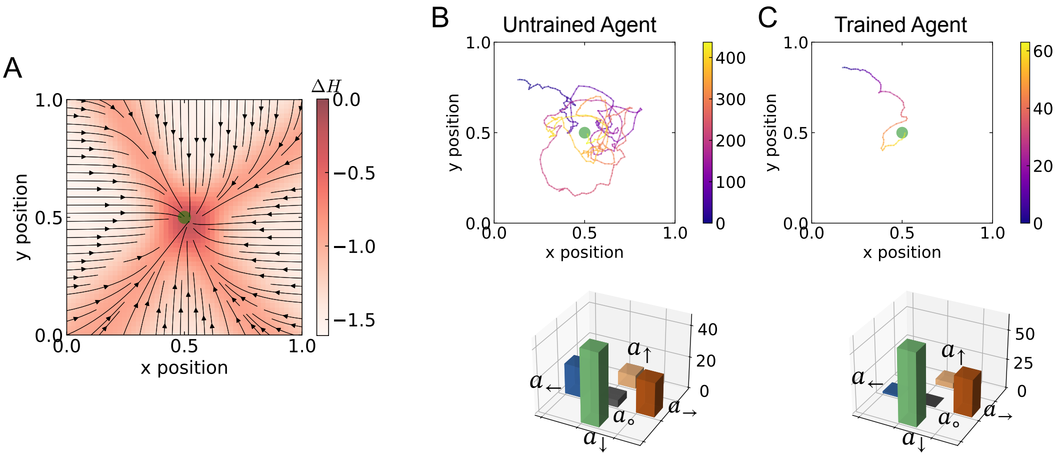

Figure S7: **Policy in dynamic-flow-perturbed environment.** **A**. Policy of an agent rained in dynamic flow-perturbed environment leads characterized by Shannon entropy (ΔH) and expected velocity field $\langle \vec{v}_{\text{A}} \rangle_{\text{flow}}$. **B**. (top) Example trajectory of an initialized agent in presence of dynamic flow exhibits significant deviation and (bottom) the corresponding distribution of the agent's action in the episode. **C**. (top) Trajectory of a trained agent in presence of dynamic flow showcasing convergence behavior and (bottom) the corresponding distribution of the agent's action in the episode.

Dynamic flow-perturbation was realized by scanning the heating laser around a closed loop that changes with time. This spatio-temporally dynamic flow leads to corresponding instantaneous advection of the agent during navigation. The absence of any temporal phase information or memory leads to the agent perceiving only net effect due to each action in its states, which over repeated interaction translates to time-averaged effect due to the flow-perturbation. The average perturbation along with the task of navigating to the target leads to a policy shown in Fig. S7A, characterized by the entropy difference (ΔH) and expected velocity field ($\langle \vec{v}_{\text{A}} \rangle_{\text{flow}}$). The policy is qualitatively similar to radially inward policy shown for an inert environment. The trajectory of an initialized untrained agent along with the distribution of its actions in the episode, shown in Fig. S7B (top) and (bottom) respectively, exhibits significant deflection during its navigation. For a trained agent, the deflection is less, as shown in Fig. S7C, and the resulting action distribution indicates radially inward motion.

S8 Caption for the supplementary videos

- Video 1: Initialized and trained agent's trajectory in inert environment
- Video 2: Policy evolution in inert and flow-perturbed environment
- Video 3: Initialized and trained agent's trajectory in flow-perturbed environment
- Video 4: Trajectory for different initial conditions in dynamic flow-field
